# Case of Dropsy

**Published:** 1803-12-01

**Authors:** 

**Affiliations:** Bath


### Case of Dropsy, *communicated by* Mr. Bush, *of Bath.*

MISS E. W. of this city, aged 29 years, was, about five years since, (she supposed from having taken cold) seized with the *Ascitæs Abdominalis,* which continued to increase for three years, notwithstanding all the medicines in general use were given by different medical gentlemen, from its commencement; until she applied, about two years and half since, to my father, who was at that time my partner. We likewise tried the use of medicine for some months with as little success. As she still continued to increase in size, and was very much reduced, I advised her to undergo an operation, which would at least render her a temporary relief; I performed the operation in November, 1801, and drew off three gallons of clear water, which was impregnated with salt; after finishing the operation in the usual way, I gave her *ferri rubigo* in form of pills bis die; twice a week I gave her an aperient; and in addition to this she took daily some diuretic medicine. She was ordered to take as little fluid as possible; and I was happy to find, that contrary to my expectation, she grew less, and has regained her former size and health. As the operation is considered a palliative, and not a radical cure, I think this case deserves notice, as some hopes may be entertained of recovering such patients lastingly by a similar treatment. The ascites was in this case a primary disease, as she had no complaint previous to its commencement, except fits, which she had been troubled with for many years, and which still continue.

